# Speech extraction from vibration signals based on deep learning

**DOI:** 10.1371/journal.pone.0288847

**Published:** 2023-10-25

**Authors:** Li Wang, Weiguang Zheng, Shande Li, Qibai Huang

**Affiliations:** 1 State Key Laboratory of Digital Manufacturing Equipment and Technology, School of Mechanical Science and Engineering, Huazhong University of Science and Technology, Wuhan, China; 2 Hubei University of Arts and Science, Xiangyang, China; 3 Hubei Innovation Center of Mobile Emergency Equipment Manufacturing, Hubei Institute of Specialty Vehicle, Suizhou, China; 4 School of Mechanical and Automotive Engineering, Guangxi University of Science and Technology, Liuzhou, China; Valahia University of Targoviste: Universitatea Valahia din Targoviste, ROMANIA

## Abstract

Extracting speech information from vibration response signals is a typical system identification problem, and the traditional method is too sensitive to deviations such as model parameters, noise, boundary conditions, and position. A method was proposed to obtain speech signals by collecting vibration signals of vibroacoustic systems for deep learning training in the work. The vibroacoustic coupling finite element model was first established with the voice signal as the excitation source. The vibration acceleration signals of the vibration response point were used as the training set to extract its spectral characteristics. Training was performed by two types of networks: fully connected, and convolutional. And it is found that the Fully Connected network prediction model has faster Rate of convergence and better quality of extracted speech. The amplitude spectra of the output speech signals (network output) and the phase of the vibration signals were used to convert extracted speech signals back to the time domain during the test set. The simulation results showed that the positions of the vibration response points had little effect on the quality of speech recognition, and good speech extraction quality can be obtained. The noises of the speech signals posed a greater influence on the speech extraction quality than the noises of the vibration signals. Extracted speech quality was poor when both had large noises. This method was robust to the position deviation of vibration responses during training and testing. The smaller the structural flexibility, the better the speech extraction quality. The quality of speech extraction was reduced in a trained system as the mass of node increased in the test set, but with negligible differences. Changes in boundary conditions did not significantly affect extracted speech quality. The speech extraction model proposed in the work has good robustness to position deviations, quality deviations, and boundary conditions.

## 1. Introduction

The extraction of speech signals is the first step in speech processing. Sound data collected by the acoustic signal hardware acquisition equipment are generally waveform data. They are processed after the acoustic signal is converted into an electrical signal, that is, time-varying sound wave information emitted by the sound source. The feature extraction from the acoustic signal plays significant role in several applications such as automotive system to assist steering, interaction between a human and machine at home, hospitals, shops etc. Mel-frequency cepstral coefficients (MFCC) features are well used for speech recognition and voice classification. Ranjan *et al*. [1] proposed that the delta-delta MFCC feature extraction technique is better than the other MFCC techniques. Koduru *et al*. [2] preprocesses the received audio samples, using filters to remove noise from the speech samples. In the next step, Mel frequency cepstrum coefficient (MFCC), Discrete wavelet transform (DWT), pitch, energy and Zero-crossing rate (ZCR) algorithms are used to extract features. These feature extraction algorithms have been validated for general emotions including anger, happiness, sadness, and neutrality.

However, acoustic signals, unlike vibration signals, are inconvenient to be measured directly in some voice monitoring cases. A case in point is the restoration of the voice signals under dynamic local recognition after the surrounding vibration signals are measured by the long-distance laser vibrations. Traditional identification methods include direct inverse [3], regularization [4], probability, and statistics [5], but they are sensitive to noises [6] and measurement point positions.

Recently, deep learning methods based on deep neural networks (DNNs) have promoted computer vision, natural language processing, and signal recognition rapidly. Zhou *et al*. [7] proposed a method for identifying shock loads of nonlinear structures based on deep recurrent neural networks. Deep recurrent neural networks mainly consist of two long short-term memory (LSTM) layers and a bidirectional LSTM (BLSTM) layer. It learns a complex inverse mapping between structural input and output through substantial dynamic responses and shock loads. Consequently, the proposed method can identify complex shock loads from the damped Duffing oscillator, the nonlinear three-degree-of-freedom system, and the nonlinear composite plate. Liu *et al*. [8] proposed a method of dynamic force reconstruction based on the artificial neural network (ANN) and Bayesian probability framework (BPF). The identified curves are consistent with the actual dynamics regarding amplitudes and regularity. Quantitative data are also measured under certainty and uncertainty within a reasonable range for engineering applications. Therefore, by using deep learning methods, vibration source signals can be extracted from vibration signals. However, there have been no attempts to extract speech signals from vibration signals yet.

Deep learning methods have been widely used in speech enhancement. Wang et al. [9] used deep neural networks (DNN) to abstract the sub-band features after the input signal was converted into a sub-band signal through a 64-channel Gammatone filter bank. The relevant speech posterior mask is acquired in this way. Then, a linear support vector machine or DNN is used to estimate the IBM of noisy speech based on the posterior mask. The performance speech separation algorithms can be enhanced with deep networks to improve the intelligibility of separated speech. Park *et al*. [10] suggested a frequency-domain speech denoise model under 2D convolutional neural networks (CNN), considering FNN’s positional inputs malfunction and massive parameters. The model is built by CNN and uses an encoder-decoder network structure. CNN, by contrast, equips its model to reduce parameters in 12-fold and still denoise perfectly. Hui *et al*. [11] introduced CNNs to improve the model’s ability to mine speech depth features. Chen *et al*. [12] applied LSTM to speech separation, which allows modeling on long sequences. Ideal results can be achieved even without future frames. Luo *et al*. [13] proposed a speech separation model called Conv-TasNet concerning the time domain. 1D convolution is used to transform the time domain signals into a hidden space for separation, and then the space is decoded into target signals in the time domain. Macartney *et al*. [14] proposed an efficient time-domain speech-denoising model based on 1D CNN. The dependence of speech (as timing-sequence signals) in the time dimension is also vital for speech denoising. A deep learning based integrated architecture called FuzyGCP has been proposed for recognizing spoken language from speech signals by Garain *et al*. [15]. The architecture combines the classification principles of deep dumb Multilayer perceptron (DDMLP), deep Convolutional neural network (DCNN) and Semi-supervised Generative Adversarial Network (SSGAN) to maximize the accuracy, and finally uses Choquet integral to apply Ensemble learning to predict the final output. However, CNN is not good at extracting timing-sequence information. Therefore, researchers applied recurrent neural networks (RNNs) that are better at processing timing-sequence information to speech denoising. Tan *et al*. [16] discovered a speech denoise model based on convolutional recursive networks (CRNs). It denoises better by combining the advantages of CNNs in extracting local information with that of LSTM in time modeling. Defossez *et al*. [17] combined the gating mechanism with CRN. They upgraded the model by adding a gating mechanism into each layer of CNN to filter noises. Since its inception, RNN has undergone continuous evolution and iteration, applying LSTM to the previously difficult problem of implementing back propagation. The simplified GRU developed by LSTM can also maintain a certain level of accuracy. However, as mentioned in a recent public paper, as a member of the CNN family, Temporal convolutional networks (TCN) [18], successfully defeated RNN in various datasets and became a promising member in the analysis of new sequence data. Tandale *et al*. [19] evaluated the gated RNN model including LSTM and GRU units, followed by the TCN architecture to develop an effective alternative model to learn the midpoint deformation behavior of complex path related shock wave loading plates.

Transformer [20] has also been applied to natural language processing and image processing, and speech-denoising models based on Transformer appear recently. Kim *et al*. [21] proposed a frequency-domain speech-denoising model based on Transformer. It promotes the Transformer in speech denoising by adding Gaussian weighting to all self-attention layers so that nearby frames have greater attention weight. Wang *et al*. [22] proposed a time-domain speech-denoising model based on a two-stage Transformer. The model extracts the local and global timing information of long-term speech sequences, which can achieve a good denoising effect. An improved Swin Transformer has been proposed for segmenting dense urban buildings from remote sensing images with complex backgrounds [23]. The original Swin Transformer was used as the backbone of the encoder, while the convolutional block attention module was used in the linear embedding and patch merging stages to focus on important features. Then, the hierarchical feature map is fused to enhance the feature extraction process, and it is input into UPerNet (as a decoder) to obtain the final segmentation map. The collapsed and non collapsed buildings were marked from remote sensing images of the Yushu and Beichuan earthquakes. Perform data augmentation for horizontal and vertical flipping, brightness adjustment, uniform and non-uniform atomization to simulate actual situations. The effectiveness and superiority of this method compared to the original Swin Transformer and several mature CNN based segmentation models were verified through ablation experiments and comparative studies.

In conclusion, deep learning has been successfully applied in signal processing, image recognition, Machine translation, speech recognition, emotion recognition, etc. There has been no research on extracting speech signals through vibration signals, when acoustic signals are inconvenient to be measured directly in some cases. The focus of this article is to extract speech signals from the acoustic vibration coupling model and verify the effectiveness of deep learning methods for such problems. Section 2 introduces the proposed model and method, two types of networks are applied to the task: fully connected, and convolutional. Section 3 analyzes how the response point positions, noises, position deviations, node quality, and boundary conditions affect extracted speech quality. Section 4 summarizes the work.

## 2. Model and method

**Fig 1:**
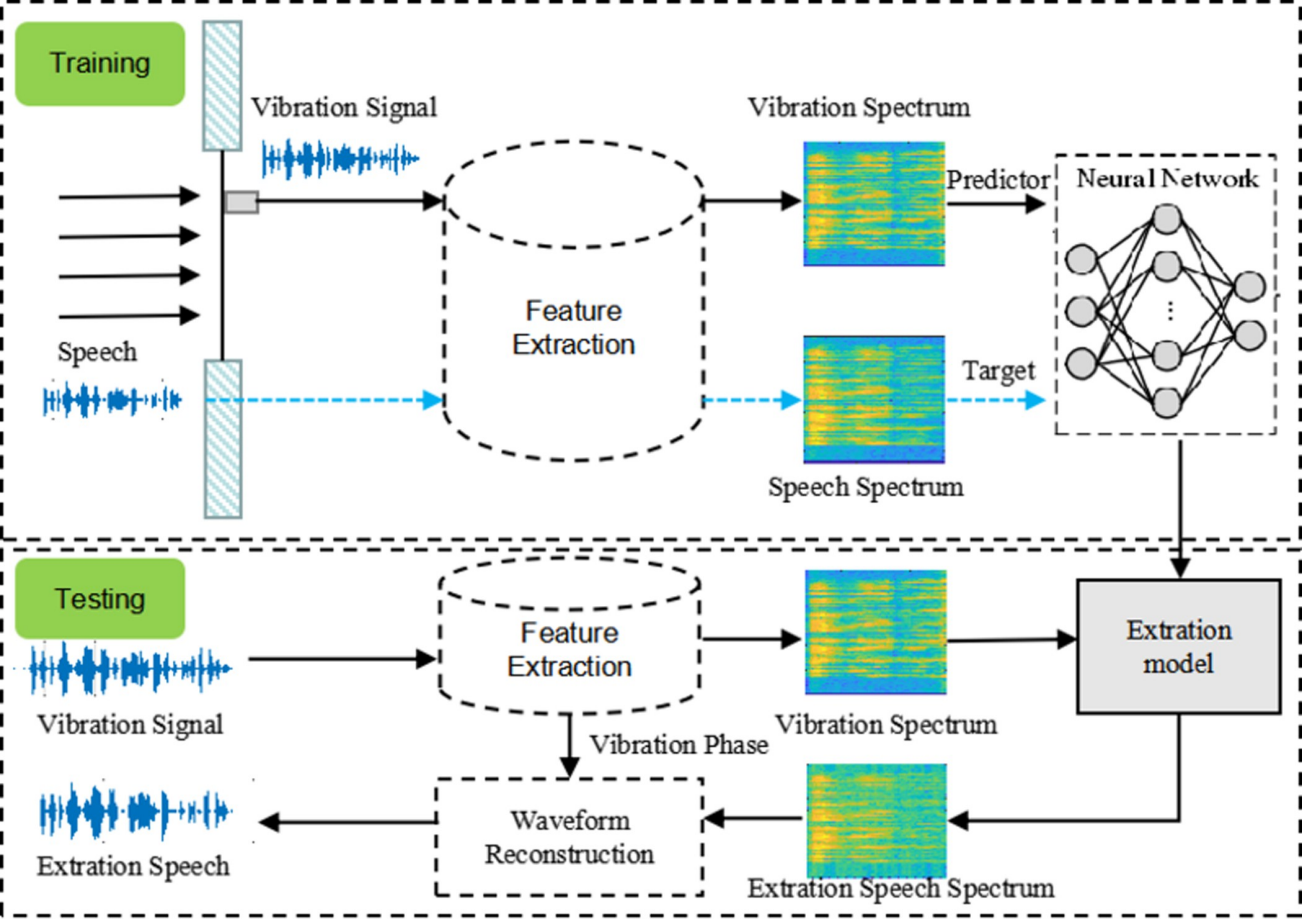
Framework of the method proposed in the work.

The main problem to be studied in the work is extracting sound signals through vibration response. Fig 1 lists the framework. It is assumed that sound waves are incident vertically on a flexible sheet, and the vibration response on the sheet is collected through a sensor. The predictor signals and the network target signals are the amplitude spectra of the vibration response signals and the clean audio signals, respectively. The output of the network is to extract the amplitude spectra of the speech signals. The regression network uses the input of the predictor variable to minimize the mean square error between its output and input targets. The amplitude spectra of the output and the phase of the vibration signals are used to convert the extracted audio back to the time domain.

### 2.1 Acoustic-structure response

A flat plate was selected to be placed between the infinitely large baffles to simplify the analysis. The vibration of the plate was caused by the sound waves acting on the plate. The vibroacoustic response was obtained by the finite element method. Plate elements were modeled by shell elements with three mobile and two rotational degrees of freedom per node [24]. The displacement field in the shell element can be expressed as follows according to the classical plate-shell theory.

$$u(x,y,z,t)\;=\;u_{0}(x,y,t)\;-\;z\frac{\partial w_{0}}{\partial x}$$ (1)

$$v(x,y,z,t)\;=\;v_{0}(x,y,t)\;-\;z\frac{\partial w_{0}}{\partial y}$$ (2)

$$w(x,y,z,t)\;=\;w_{0}(x,y,t)$$ (3)

where u, v, and w are the displacements in the x, y, and z directions, respectively, while u_0_, v_0_, and w_0_ are the displacements on the neutral surface. Its matrix form is

$$\left\{u\}=[H]\{\bar{u}\right\}$$ (4)

where $\{u\}={\left\{\begin{array}{c} u\;v\;w \end{array}\right\}}^{T},\;\{\bar{u}\}=\left\{\begin{array}{c} u_{0}\;v_{0}\;w_{0}\;\frac{\partial w_{0}}{\partial x}\;\frac{\partial w_{0}}{\partial y} \end{array}\right\},$

$$[H]=\left[\begin{array}{ccccc} 1 & 0 & 0 & -z & 0\\ 0 & 1 & 0 & 0 & -z\\ 0 & 0 & 1 & 0 & 0 \end{array}\right]$$ (5)

The displacement on the neutral surface can be expressed by the unit interpolation function as follows:

$$u_{0}(x,y)\;=\overset{}{\underset{}{\;\Sigma_{i=1}^{4}}}u_{0i}(t)\psi_{i}(x,y)$$ (6)

$$v_{0}(x,y)\;=\overset{}{\underset{}{\;\Sigma_{i=1}^{4}}}v_{0i}(t)\psi_{i}(x,y)$$ (7)

$$w_{0}(x,y,t)\;=\;\Sigma_{i=1}^{4}\left[w_{0i}(t)g_{i1}(x,y)\;+\;\frac{\partial w_{0i}(t)}{\partial x}g_{i2}(x,y)\;+\;\frac{\partial w_{0i}(t)}{\partial y}g_{i3}(x,y)\right]$$ (8)

where ψ_i_(i = 1, 2, 3, and 4) is the linear interpolation function, while g_ij_ (j = 1, 2, and 3) is the non-conforming Hermite cubic interpolation function, that is

$$\psi_{i}\;=\;\frac{1}{4}\left(1\;+\;\xi \xi_{i}\right)\left(1\;+\;\eta \eta_{i}\right)$$ (9)

$$g_{i1}\;=\;\frac{1}{8}\left(1\;+\;\xi \xi_{i}\right)\left(1\;+\;\eta \eta_{i}\right)\left(2\;+\;\xi \xi_{i}\;+\;\eta \eta_{i}\;-\;\xi^{2}\;-\;\eta^{2}\right)$$ (10)

$$g_{i2}\;=\;\frac{1}{8}\xi_{i}{\left(1+\xi \xi_{i}\right)}^{2}\left(\xi \xi_{i}\;-\;1\right)\left(1\;+\;\eta \eta_{i}\right)a$$ (11)

$$g_{i3}\;=\;\frac{1}{8}\eta_{i}{\left(1\;+\;\eta \eta_{i}\right)}^{2}\left(\eta \eta_{i}\;-\;1\right)\left(1\;+\;\xi \xi_{i}\right)b$$ (12)

Eq (4) can be written in the following form according to Eqs (6–12):

$$\{u\}\;=\;\left[N_{s}\right]\left\{u_{e}\right\}$$ (13)

where

$$\left[N_{s}\right]\;=\;[H][N]$$ (14)

$$[N]\;=\;\left[\begin{array}{c} N_{1}\;N_{2}\;N_{3}\;N_{4} \end{array}\right]$$ (15)

$$\left[N_{i}\right]\;=\;\left[\begin{array}{ccccc} \psi_{i} & 0 & 0 & 0 & 0\\ 0 & \psi_{i} & 0 & 0 & 0\\ 0 & 0 & g_{i1} & g_{i2} & g_{i3}\\ 0 & 0 & \frac{\partial g_{i1}}{\partial x} & \frac{\partial g_{i2}}{\partial x} & \frac{\partial g_{i3}}{\partial x}\\ 0 & 0 & \frac{\partial g_{i1}}{\partial y} & \frac{\partial g_{i2}}{\partial y} & \frac{\partial g_{i3}}{\partial y} \end{array}\right]$$ (16)

It can be obtained from the relation between strain and displacement that

$$\left\{\begin{array}{c} \varepsilon_{x}\\ \varepsilon_{y}\\ \gamma_{xy} \end{array}\right\}\;=\;\left\{\begin{array}{c} \frac{\partial u}{\partial x}\\ \frac{\partial v}{\partial y}\\ \frac{\partial u}{\partial y+\frac{\partial v}{\partial x}} \end{array}\right\}$$ (17)

Eq (13) is substituted into the above equation to obtain

$$\{\varepsilon \}\;=\;\left[B_{s}\right]\left\{u_{e}\right\}$$ (18)

where [*B*_*s*_] can be obtained by differentiating [*N*_*s*_]. The mass matrix and stiffness matrix of the shell element can be written as follows from the virtual work principle.

$$\left[m^{\left(1),(3\right)}\right]\;=\;\int_{-1}^{1}\int_{-1}^{1}\int_{h^{\left(1),(3\right)}}\rho^{\left(1),(3\right)}{\left[N_{s}\right]}^{T}\left[N_{s}\right]|J|dzd\xi d\eta$$ (19)

$$\left[k^{\left(1),(3\right)}\right]\;=\;\int_{V_{e}^{\left(1),(3\right)}}{\left[B_{s}\right]}^{T}\left[E_{s}^{\left(1),(3\right)}\right]\left[B_{s}\right]dV$$ (20)

where *J* is the element Jacobian matrix; [*E*_*s*_] the shell element stiffness matrix; *V*_*e*_ the unit volume. The unit matrix is assembled into an overall matrix (Eq (21))

$$[M]\{\ddot{u}\}\;+\;[K]\{u\}\;=\;f$$ (21)

where [*M*] is the overall mass matrix; [*K*]the overall stiffness matrix; *f* the force vector.

### 2.2 Verification

Part of the Mozilla Universal voice dataset [25] is used to train and test deep learning networks. The dataset contains 48-kHz recordings of subjects dictating short sentences. The clean audio signals are first downsampled to 8 kHz to reduce computational load on the network because speech is usually lower than 4 kHz. Then, the Newmark method is used to solve the dynamic response, with a step length of 1/8000 s.

The predictor signals and the network target signals transform the vibration response or pure speech signals into the frequency domain using short-term Fourier transform (STFT), with a window length of 128 samples, an overlap rate of 75%, and a Hamming window. The size of the spectral vector can be reduced to 65 by discarding the frequency samples corresponding to the negative frequency. Since time-domain speech signals are real, they will not cause any information losses. The input of the predictor variable consists of 8 consecutive audio signal STFT vectors, so each STFT output estimate is calculated based on the current audio STFT and 7 previous audio STFT vectors.

Firstly, A network composed of fully connected layers is used to extract audio in the work, and the input size is specified as an image of 65×8. Two hidden fully connected layers are defined, each with 2,048 neurons. Since it is a purely linear system, there is a rectified linear unit (ReLU) layer behind each hidden fully connected layer. The batch normalization layer normalizes the mean and standard deviation of the output. A fully connected layer containing 65 neurons is added, followed by a regression layer. The inverse STFT transform is performed through the inverse short-term Fourier transform (ISTFT) and the phase of the STFT vector of the vibration signals is used to reconstruct the time domain speech signals in the test set.

Then, consider using a convolutional layer instead of a fully connected network [10]. The 2D convolutional layer applies a sliding filter to the input. This layer calculates the weight and input dot product by moving the filter vertically and horizontally along the input, and then adds bias terms to convolution the input. Convolutional layers typically consist of fewer parameters than fully connected layers. Define the layers of a fully convolutional network described in [10], including 16 convolutional layers. The first 15 convolutional layers are a group of 3 layers, repeated 5 times, with filter widths of 9, 5, and 9, and filter numbers of 18, 30, and 8, respectively. The last convolutional layer has a filter width of 129 and one filter. In this network, convolution is performed only in one direction (along the frequency dimension), and for all layers except the first layer, the filter width along the time dimension is set to 1.Similar to the fully connected network, convolutional layers are followed by ReLu and batch normalization layers.

**Fig 2:**
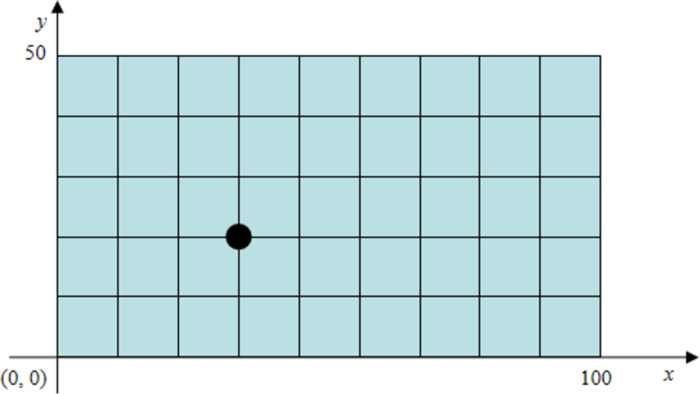
Rectangle plate.

The physical parameters of the rectangle plate are list in Table 1. The boundary conditions are taken as fixed at four edges, and 10 *×* 5 elements are used in the FE model as shown in Fig 2. Force is vertically incident according to the sound and acts evenly on the node, regardless of the influences of plate sound radiations and sound pressures.

**Table 1:** Physical parameters of the rectangle plate.

Physical parameters	Value
Length	100 mm
Weight	50 mm
Height	0.2 mm
Complex modulus of elasticity	201 × (1+0.02j) GPa
Poisson ratio	0.27
Density	7900 kg/m³

**Fig 3:**
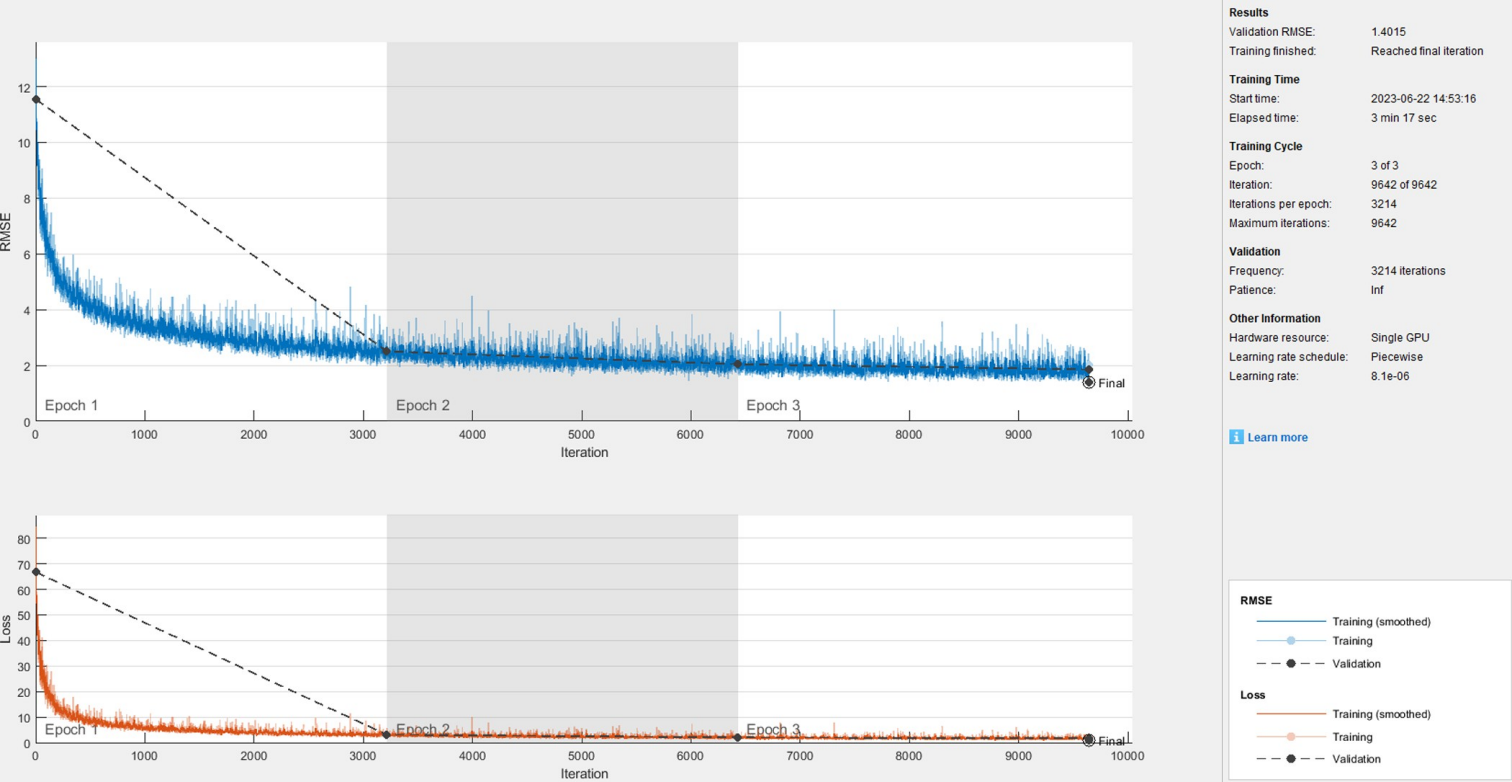
Training process using fully connected layers.

**Fig 4:**
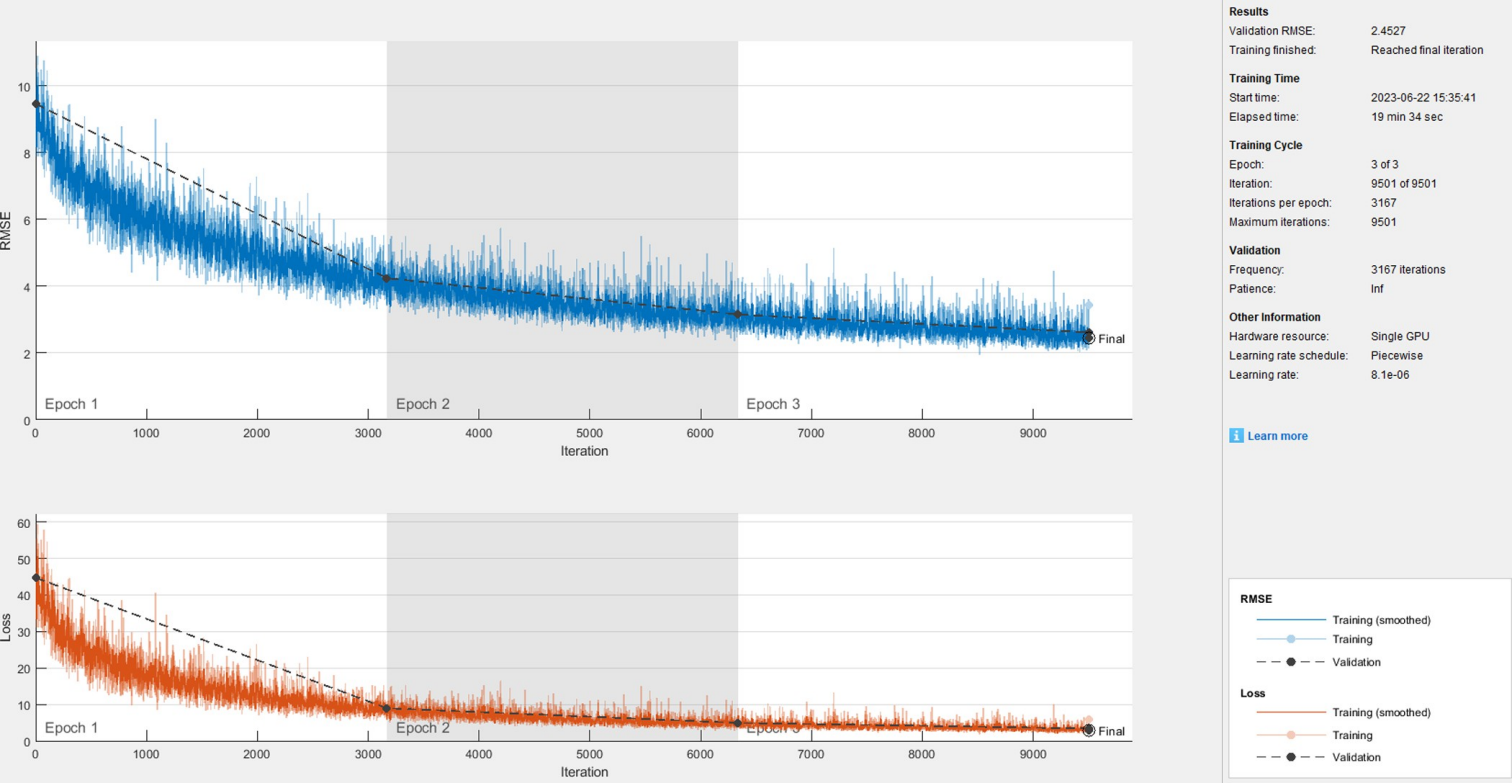
Training process using convolutional layers.

When a node with a position of (30, 20 mm) is selected as the vibration response point as shown in Fig 2, the corresponding clean speech, vibration response, and extracted response are obtained by using fully connected layers and convolutional layers. The training process is shown in Figs 3 and 4. We can find that the rate of convergence is faster and the training time is shorter (3min17sec for fully connected layers and 19min34sec for convolutional layers) using fully connected layers than using convolutional layers.

**Fig 5:**
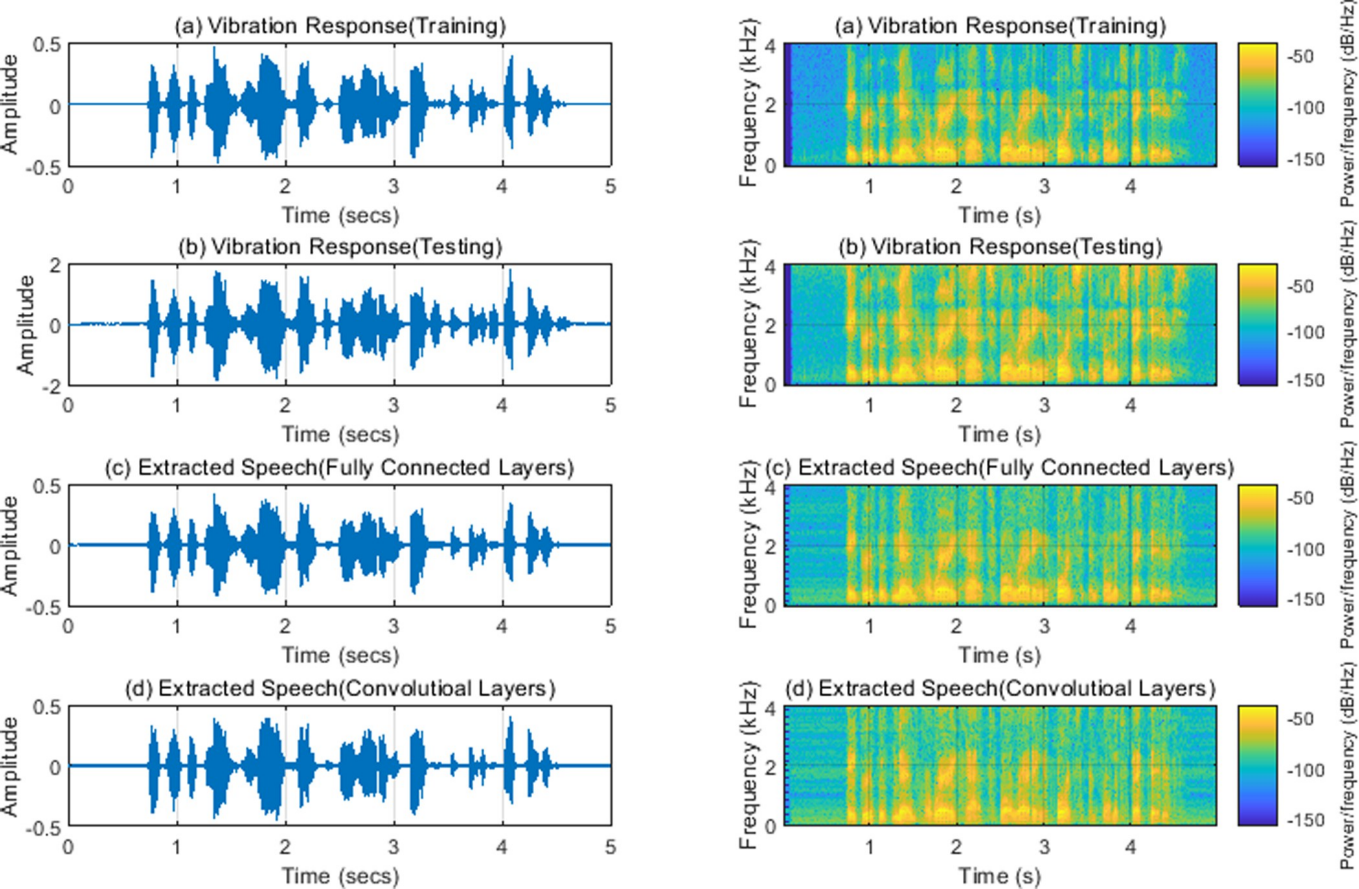
(a) Clean speech; (b) Vibration response; (c) Extracted speech(Fully Connected Layers); (d)Extracted speech(Convolutional Layers). Note: Left: time domain; Right: spectrogram.

The extracted speech in time domain and spectrogram is shown in Fig 5. The method proposed in the work can extract and reconstruct the speech signals from the vibration response. The widely used objective evaluation indices of speech quality include perceptual evaluation of speech quality (PESO) [26], MOS predictor of speech distortion (CSIG), MOS predictor of intrusiveness of background noise (CBAK), and MOS predictor of overall processed speech quality (COVL) [27]. These objective indices have a high correlation degree with people’s subjective sense of hearing and can better measure speech quality. PESQ is an objective speech quality evaluation index launched by the International Telecommunication Union with scores ranging from -0.5 to 4.5. The higher the score, the higher the speech quality. CSIG, CBAK, and COVL are complex objective evaluation indices, which can obtain a better evaluation of speech quality through a linear combination of other objective evaluation indices. Their score distribution is between 1 and 5. The higher the score, the better the speech quality.

CSIG assesses speech quality from the perspective of signal distortion. CSIG is linearly weighted by PESQ, Log-LikelihoodRatio (LLR), and Weighted Spectral Slope (WSS) (Eq (22)). CBAK evaluates speech quality from the perspective of background noise interference. CBAK is a linear weighting of PESQ, WSS, and Segment Signal-Noise ratio (SegSNR) (Eq (23)). COVL reflects the overall quality of the signal, which is also obtained by the linear weighting of PESQ, LLR, and WSS. Eq (24) lists the calculation method. Detailed calculations of LLR, WSS, and SegSNR refer to the studies of Hu *et al*. [27].


CSIG=3.093−1.029⋅LLR+0.603⋅PESQ−0.009⋅WSS (22)

CBAK=1.634+0.478⋅PESQ−0.007⋅WSS+0.063⋅SegSNR (23)

CVOL=1.594+0.805⋅PESQ−0.512⋅LLR−0.007⋅WSS (24)

The objective evaluation of extracted speech using different layers is shown in Table 2. We can find that the speech quality extracted using Fully Connected layers is better than that using Convolutional layers. Because the Fully Connected network prediction model has faster Rate of convergence and better quality of extracted speech, this method will be used to analyze the impact of other factors in the subsequent research of this paper.

**Table 2:** Objective evaluation of extracted speech using different layers.

Layer type	LLR	segSNR	WSS	PESQ	CSIG	CBAK	COVL
Fully Connected	0.4946	4.6250	16.5306	3.1158	4.3141	3.2990	3.7333
Convolutional	0.8134	2.6092	30.1715	2.4729	3.4755	2.7691	2.9568

## 3. Discussion

### 3.1 Node position

This section examines the influence of the response node position on speech extraction because the vibration response is related to the structure position. The node at 1/4 of the structure is selected due to the symmetry of the structure. Each node is trained separately and its objective evaluation indices of speech are compared (Table 3). The speech extraction results of all nodes are quite good.

**Table 3:** Objective evaluation of extracted speech at different nodes.

Node (mm)	LLR	segSNR	WSS	PESQ	CSIG	CBAK	COVL
(10,10)	0.3830	4.6686	14.5099	3.1735	4.4819	3.3435	3.8510
(10,20)	0.4373	5.3069	20.3484	3.2677	4.4303	3.3879	3.8582
(20,10)	0.5343	4.7186	21.8668	3.1226	4.2293	3.2708	3.6811
(20,20)	0.4181	4.4953	15.7245	3.1688	4.4321	3.3218	3.8208
(30,10)	0.3926	5.1754	19.8810	3.1612	4.4163	3.3319	3.7986
(30,20)	0.4946	4.6250	16.5306	3.1158	4.3141	3.2990	3.7333
(40,10)	0.4833	4.9220	17.0045	3.1888	4.3655	3.3493	3.7945
(40,20)	0.4499	5.1718	21.4171	3.1796	4.3546	3.3297	3.7733
(50,10)	0.5053	4.8731	20.7326	3.2365	4.3381	3.3429	3.7955
(50,20)	0.4010	5.0085	21.6344	3.1710	4.3978	3.3138	3.7899

Due to the correlation between objective evaluation indicators of speech quality, we have chosen PESQ, CSIG, CBAK and COVL as evaluation indicators in observations of Analysis of Variance (ANOVA). the results of ANOVA indicate that there is no significant difference (P>0.05)in the speech extraction effect of different nodes by considering the sample selection error in the training process, which can be seen in Tables 4 and 5.

**Table 4:** Summary of variance for different node position.

Node (mm)	Observations	Sum	Average	Variance
(10,10)	4	14.8499	3.712475	0.345946869
(10,20)	4	14.9441	3.736025	0.279154609
(20,10)	4	14.3038	3.57595	0.24552183
(20,20)	4	14.7435	3.685875	0.324991223
(30,10)	4	14.708	3.677	0.315498233
(30,20)	4	14.4622	3.61555	0.283930377
(40,10)	4	14.6981	3.674525	0.277846843
(40,20)	4	14.6372	3.6593	0.278395447
(50,10)	4	14.713	3.67825	0.252250757
(50,20)	4	14.6725	3.668125	0.306644743

**Table 5:** Node position analysis of variance.

Variance Source	SS	df	MS	F	P-value	F crit
Between Group	0.0738	9	0.0082	0.0282	1.0000	2.2107
Inner Group	8.7305	30	0.2910			
Total	8.8044	39				

### 3.2 Noises

**Fig 6:**
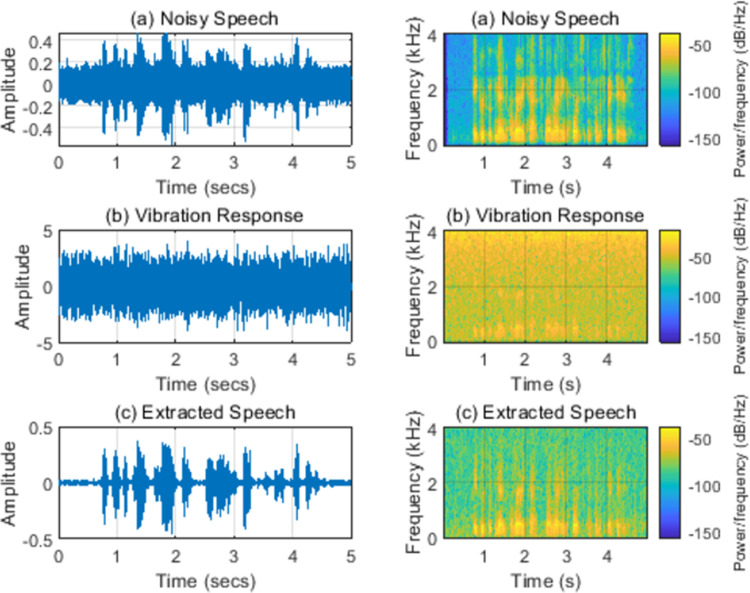
(a) Noisy speech (0 dB); (b) Vibration response; (c) Extracted speech. Note: Left: time domain; Right: spectrogram.

Speech signals and vibration response signals will inevitably be disturbed by noise in actual situations. The nodes at (30, 20 mm) positions are selected as the research objects without losing generality in this section. Table 6 shows the objective evaluation of extracted speech quality when white noises with the signal-to-noise ratios (SNR) of 5, 0, and -5dB are added to the pure speech signals. The increased speech noise signals will reduce the quality of extracted speech. Fig 4 shows the time domain and time-frequency domain spectrum of the extracted speech when the SNR equals 0 dB. Speech signals containing noise have a great impact on vibration response signals, which affects the extraction quality of pure speech by comparing Figs 5(B) and 6(B). However, it can still extract pure speech, which shows good noise robustness.

**Table 6:** Speech signals with noises.

SNR (dB)	LLR	segSNR	WSS	PESQ	CSIG	CBAK	COVL
5	0.9469	1.1788	44.2540	**2.3872**	**3.1599**	**2.5396**	**2.7212**
0	1.1029	-0.2096	49.5270	2.1886	2.8321	2.3203	2.4444
-5	1.2240	-1.6586	56.8400	1.8908	2.4621	2.0355	2.0915

The collected vibration response signal may have noise interference, affected by the external environment or sensors. Table 7 shows the objective evaluation of the extracted speech quality when 5, 0, and -5dB white noise are added to the vibration response signals separately under pure speech. Similar to noisy speech, the vibration response signal alone reduces the quality of speech extraction as the noise increases. Its impact is less than that of noise speech by comparing Table 6.

**Table 7:** Vibration response signals with noises.

SNR (dB)	LLR	segSNR	WSS	PESQ	CSIG	CBAK	COVL
5	0.8065	1.2161	44.9956	**2.5107**	**3.3721**	**2.5958**	**2.8872**
0	0.9521	-0.2724	51.7967	2.3135	3.0421	2.3601	2.6063
-5	1.1447	-1.7987	59.7056	2.1203	2.6563	2.1163	2.2968

**Fig 7:**
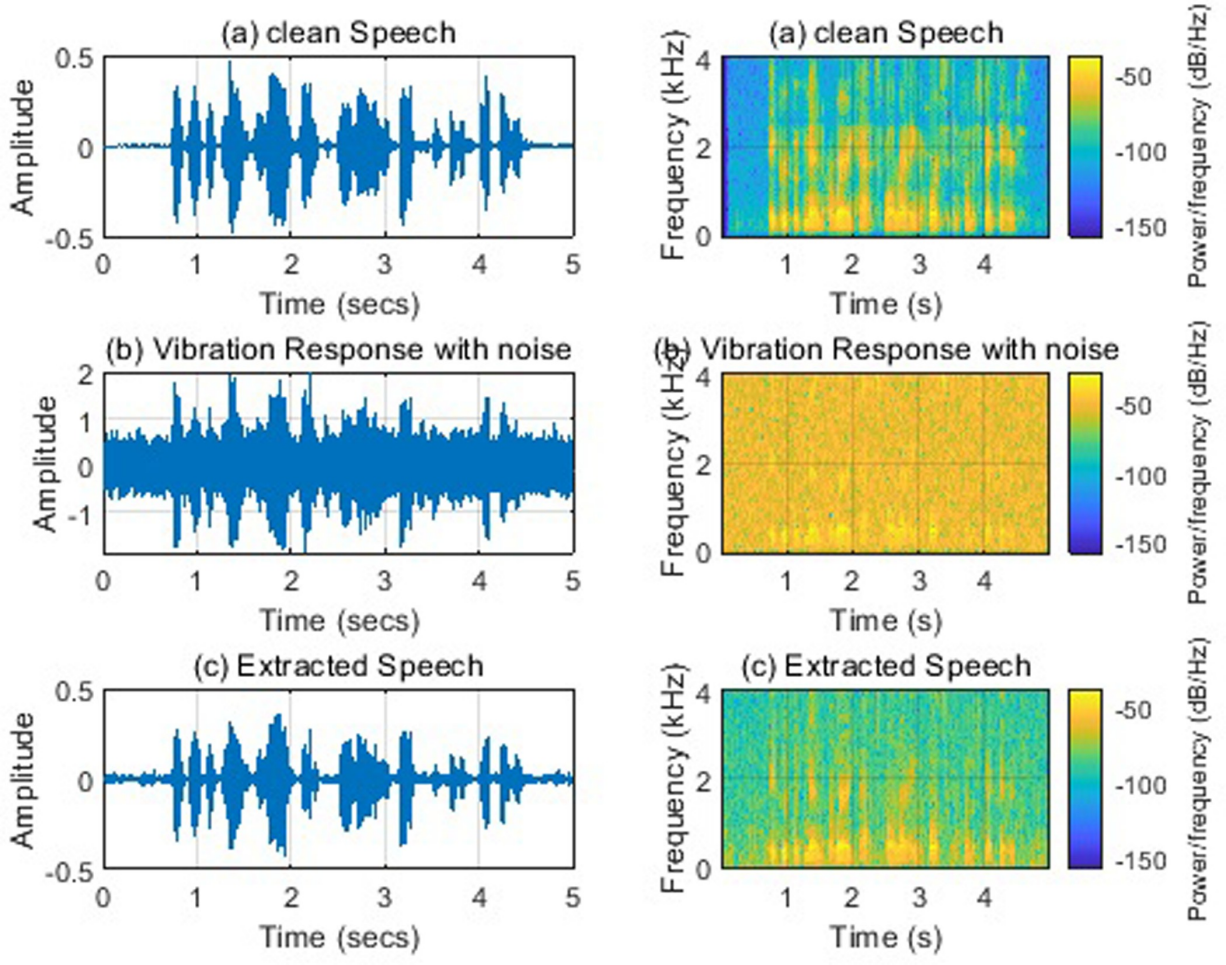
(a) Clean speech; (b) Vibration response with noises (0dB); (c) Extracted speech. Note: Left: time domain; Right: spectrogram.

Fig 7 shows the time domain and time-frequency domain spectrum of speech extracted when the noise is added to vibration response signals with SNR = 0 dB. The impact of noise on the vibration signals alone is less than that of noise on the speech alone. The noise of the vibration response will be amplified by the speech signal through the acoustic vibration system, which will reduce the quality of the extracted noise by comparing Fig 6(B).

Table 8 shows the objective evaluation of the extracted speech quality when 5-dB white noises are added to the speech signals; 10-, 5-, and 0-dB white noises are added to the vibration response signals, respectively. Increasing the noise of the vibration signals further reduces extracted speech quality since the noises of the speech signals are amplified by the acoustic vibration system to affect the vibration signals.

**Table 8:** Objective evaluation of extracted speech when the speech signals (5 dB) and the vibration response signals contain noises.

SNR (dB)	LLR	segSNR	WSS	PESQ	CSIG	CBAK	COVL
10	1.0412	-0.1776	50.8575	**2.2210**	**2.9032**	**2.3285**	**2.4929**
5	1.1254	-1.3712	55.7216	2.0767	2.6857	2.1502	2.2995
0	1.3227	-2.8447	62.4894	1.7760	2.2404	1.8663	1.9090

**Fig 8:**
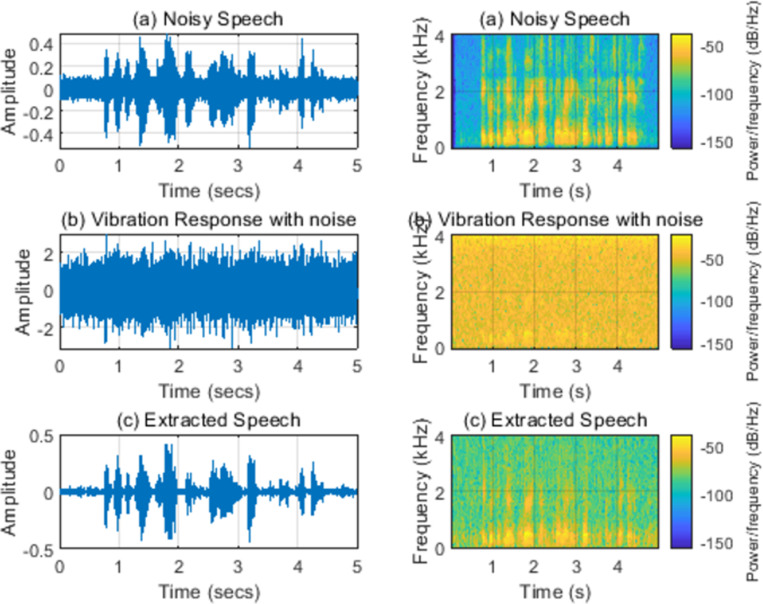
(a) Noisy speech (SNR = 5 dB); (b) Vibration response with noises (SNR = 5 dB); (c) Extracted speech. Note: Left: time domain; Right: spectrogram.

Fig 8 shows the time domain and time-frequency domain spectra of speech extracted when 5-dB white noises are added to the speech signals and the response signals, respectively. The addition of composite noise has caused a sharp decrease in extracted speech quality. Its extraction quality needs to be further enhanced although this method still has certain speech extraction capabilities.

### 3.3 Location deviation

The location of the vibration response sensor may change due to sensor sliding or laser vibration sensor positioning deviation in practical applications, which results in inconsistencies between the sensor location during training and the prediction. This section discusses the impact of location deviation on the prediction system. The nodes at the (30, 20 mm) position are still selected as the vibration response nodes during training, and the locations of other nodes are selected for comparison during the test set. The quality of extracted speech is axially symmetric concerning the geometric center line of the plate structure (Table 9) because the structure, boundary conditions, and excitation are all axially symmetric. Its vibration mode is also symmetric with the same amplitude from the structural modal analysis. As a result, the speech quality of the symmetrical points in the test set has symmetry.

**Table 9:** Extract objective evaluation of speech quality when the locations of the test point change.

Location	LLR	segSNR	WSS	PESQ	CSIG	CBAK	COVL
(10,20)	0.7524	0.1032	22.1355	2.8277	3.8247	2.8372	3.3302
(20,20)	0.5542	3.1931	18.3157	3.0255	4.1823	3.1531	3.6176
(30,20)	0.4946	4.6250	16.5306	3.1158	4.3141	3.2990	3.7333
(40,20)	0.4716	4.2709	15.8735	3.1506	4.3646	**3.2979**	3.7776
(50,20)	0.4659	4.0481	15.7189	**3.1604**	**4.3778**	3.2897	**3.7895**
(60,20)	0.4716	4.2709	15.8735	3.1506	4.3646	**3.2979**	3.7776
(70,20)	0.4946	4.6250	16.5306	3.1158	4.3141	3.2990	3.7333
(80,20)	0.5542	3.1931	18.3157	3.0255	4.1823	3.1531	3.6176
(90,20)	0.7524	0.1032	22.1355	2.8277	3.8247	2.8372	3.3302
(30,10)	0.6626	1.0721	20.5718	2.9127	3.9823	2.9498	3.4554
(30,30)	0.4946	4.6250	16.5306	**3.1158**	**4.3141**	**3.2990**	**3.7333**
(30,40)	0.6626	1.0721	20.5718	2.9127	3.9823	2.9498	3.4554
(10,10)	0.9634	-0.9212	24.4114	2.5871	3.4420	2.6417	3.0125
(20,10)	0.7317	0.2802	21.7453	2.8504	3.8631	2.8619	3.3617
(40,10)	0.6373	1.4363	20.1245	2.9383	4.0279	2.9881	3.4922
(50,10)	0.6310	1.5337	20.0256	**2.9451**	**4.0393**	**2.9982**	**3.5015**

The closer the test point is to the symmetry center, the better the extracted speech quality. It is even better than a situation where the training point and the prediction point are consistent. Extracted speech quality decreases to a certain extent at the test points close to the boundary of the structure. In conclusion, the location deviation does not have much impact on speech quality within a certain range of the training points, which provides conditions for actual engineering applications.

### 3.4 Added mass

There are still impurities or mass changes in the vibration sensor, which changes the mass matrix of the structure in the actual application process of the plate structure. This section examines the sensitivity of extracted speech quality when the test node mass changes without changing the prediction model. Similarly, nodes at (30, 20 mm) are selected as the test nodes. The mass of the node remains unchanged during the training, but the node mass is gradually increased during testing. The objective evaluations of extracted speech quality are shown when the mass of the test node are increased by 1, 5, and 10 times of element mass, respectively (Table 10). Extracted speech quality decreases as the node mass increases, but the impact is subtle. Besides, it has good robustness.

**Table 10:** Changes in node mass—an objective evaluation of extracted speech.

Mass multiple	LLR	segSNR	WSS	PESQ	CSIG	CBAK	COVL
1	0.4952	4.7467	16.5916	**3.1099**	**4.3094**	**3.3034**	**3.7278**
5	0.5051	4.8369	16.7147	3.0895	4.2858	3.2985	3.7054
10	0.5245	4.4834	17.1646	3.0608	4.2444	3.2593	

### 3.5 Boundary conditions

The boundary conditions of the plate may change in actual situations, such as boundary loosening. It is assumed in this section that the boundaries at (100, 10), (100, 20), (100, 30), and (100, 40) change from a clamped support to a free boundary. Table 11 shows that the vibration response of nodes at (30, 20 mm) position is taken as the training set under the condition of clamped support on four sides. The objective evaluation of speech extracted from 3 different nodes can get better results at different test points in the case of boundary changes in the test set. The speech quality is no longer symmetrical. The closer it is to the free boundary, the better the speech recognition quality. The lower the structural flexibility, the better the speech extraction quality combined with Section 3.3. The speech extraction model proposed in the work is also insensitive to boundary changes.

**Table 11:** Objective evaluation of extracted speech when boundary changes.

Node (mm)	LLR	segSNR	WSS	PESQ	CSIG	CBAK	COVL
(30, 20)	0.4954	4.5856	16.5622	3.0982	4.3024	3.2879	3.7185
(50, 20)	0.4333	5.0393	17.2228	3.1371	4.3838	**3.3305**	3.7770
(80, 20)	0.4061	3.8049	15.9945	**3.1868**	**4.4528**	3.2850	

## 4. Conclusion

The vibration signals of the acoustic vibration system were collected in the work for deep learning training to obtain speech signals. It is found that the Fully Connected network prediction model has faster Rate of convergence and better quality of extracted speech than that using Convolutional layers. The simulation results showed that better speech quality was obtained in many cases. The location of the vibration response point had little effect on the speech recognition quality during training. The noises of speech signals had a greater influence on the quality of speech extraction than that of vibration signals. The effect of speech quality extraction was poor when both had high noise degrees, but it still had a certain extraction ability. The quality of speech extraction was less affected by the deviation of the vibration response location during training and testing, and it had good robustness within a certain range. The speech extraction quality was also symmetrical. The lower the structural flexibility, the better the speech extraction quality.

Increased node mass reduced extracted speech quality during the test, but it had little effect. Meanwhile, extracted speech quality did not change significantly if the boundary conditions changed during the test, and the location deviation did not have much impact. Besides, the lower the structural flexibility, the better the speech extraction quality. The speech extraction model proposed in the work had good robustness to location deviation, mass deviation, and boundary conditions from the above analysis. It had better advantages and engineering application prospects compared with traditional pattern-recognition methods. The effectiveness of the proposed method will be verified by experiments in the next step, and the training model will be further optimized to reduce the influence of speech and vibration signal noises on extracted speech quality.

## Supporting information

S1 File (ZIP)
